# *Lactobacillus brevis* BGZLS10-17 and *Lb. plantarum* BGPKM22 Exhibit Anti-Inflammatory Effect by Attenuation of NF-κB and MAPK Signaling in Human Bronchial Epithelial Cells

**DOI:** 10.3390/ijms23105547

**Published:** 2022-05-16

**Authors:** Marija Stankovic, Katarina Veljovic, Nikola Popovic, Snezana Kojic, Sofija Dunjic Manevski, Dragica Radojkovic, Natasa Golic

**Affiliations:** 1 Laboratory for Molecular Biology, Institute of Molecular Genetics and Genetic Engineering, University of Belgrade, 11042 Belgrade, Serbia; snezanakojic@imgge.bg.ac.rs (S.K.); sofijadunjic@imgge.bg.ac.rs (S.D.M.); dada@imgge.bg.ac.rs (D.R.); 2 Laboratory for Molecular Microbiology, Institute of Molecular Genetics and Genetic Engineering, University of Belgrade, 11042 Belgrade, Serbia; katarinav@imgge.bg.ac.rs (K.V.); popovicnikola@imgge.bg.ac.rs (N.P.); natasag@imgge.bg.ac.rs (N.G.)

**Keywords:** lactic acid bacteria, bronchial epithelial cells, immunomodulatory, anti-inflammatory, NF-κB signaling, MAPK signaling

## Abstract

Bronchial epithelial cells are exposed to environmental influences, microbiota, and pathogens and also serve as a powerful effector that initiate and propagate inflammation by the release of pro-inflammatory mediators. Recent studies suggested that lung microbiota differ between inflammatory lung diseases and healthy lungs implicating their contribution in the modulation of lung immunity. Lactic acid bacteria (LAB) are natural inhabitants of healthy human lungs and also possess immunomodulatory effects, but so far, there are no studies investigating their anti-inflammatory potential in respiratory cells. In this study, we investigated immunomodulatory features of 21 natural LAB strains in lipopolysaccharide (LPS)-stimulated human bronchial epithelial cells (BEAS-2B). Our results show that several LAB strains reduced the expression of pro-inflammatory cytokine and chemokine genes. We also demonstrated that two LAB strains, *Lactobacillus brevis* BGZLS10-17 and *Lb. plantarum* BGPKM22, effectively attenuated LPS-induced nuclear factor-κB (NF-κB) nuclear translocation. Moreover, BGZLS10-17 and BGPKM22 reduced the activation of p38, extracellular signal-related kinase (ERK), and c-Jun amino-terminal kinase (JNK) signaling cascade resulting in a reduction of pro-inflammatory mediator expressions in BEAS-2B cells. Collectively, the LAB strains BGZLS10-17 and BGPKM22 exhibited anti-inflammatory effects in BEAS-2B cells and could be employed to balance immune response in lungs and replenish diminished lung microbiota in chronic lung diseases.

## 1. Introduction

The interior of the airways is lined with epithelial cells acting as a defensive barrier [1]. Bronchial epithelial cells are the site of contact with environmental factors, such as microorganisms, gases, and allergens, and have a crucial role in maintaining normal airway structure and function [1,2]. Although long considered sterile, airways are characterized by a relatively low density of commensal bacteria that constitute lung microbiota. The microbiota of healthy human lungs are dominated by Firmicutes, Bacteroidetes, Proteobacteria, Fusobacteria, and Actinobacteria phyla showing substantial interindividual and intraindividual variability [3,4]. Exposure of the airways to the commensal bacteria in early life has a crucial role in priming and shaping the immune system and prevents the development of early life asthma [4,5,6].

The epithelial barrier also mediates the clearance of microbial pathogens and the adaptation to the commensal bacteria by immune responses that minimize inflammation and maintain tissue homeostasis [2]. Bronchial epithelial cells recognize molecules that are specific to microbes, namely pathogen-associated molecular patterns (PAMPs), via the expression of pattern recognition receptors (PRRs), such as Toll-like receptors (TLRs), and release cytokines and chemokines that are important in innate and adaptive immune processes [1]. The activation of TLR4, which recognizes lipopolysaccharides (LPSs) from Gram-negative bacteria, leads to the activation of nuclear factor-κB (NF-κB) and mitogen-activated protein kinases (MAPKs), including extracellular signal-related kinases (ERKs), c-Jun amino-terminal kinases (JNKs), and p38 as the main kinases, subsequently leading to the transcription of the pro-inflammatory genes *interleukin* (*IL*)-*1β*, *IL-6*, and *IL-8* (*CXCL-8*) and the *tumor necrosis factor* (*TNF*) [7,8]. Inflammation plays an important role in destroying the invading microbes and protecting the organisms, but unregulated inflammation is an underlying cause of many chronic lung diseases.

Chronic inflammatory lung diseases such as chronic obstructive pulmonary disease (COPD), neutrophilic asthma, cystic fibrosis (CF), bronchiectasis, and pulmonary fibrosis are characterized by activated immune and structural lung cells producing a variety of pro-inflammatory mediators, leading to excessive and self-perpetuating airway inflammation and tissue destruction [9,10,11,12,13]. Lung inflammation is increased with the progress of disease, and it is even further amplified during acute exacerbations, which are usually triggered by pathogen expansion [9,10,11,12,13]. Exacerbations are associated with significant morbidity and mortality and are followed by the activation of NF-κB and MAPK signaling cascades and the expression of multiple inflammatory genes [7,14,15]. Moreover, changes in the structure, abundance, and diversity of lung microbiota are associated with the chronic inflammatory lung diseases [4,16,17,18,19,20,21]. Lung microbiota dysbiosis is also correlated with disease severity and exacerbations [3,21].

Hence, it has been presumed that the alteration of lung microbiota contributes to the impairment in lung immunity and pulmonary inflammation and promotes infections, susceptibility, and exacerbation of chronic lung diseases leading to the destruction of the lungs [4,17,18,19,20]. However, it is still unclear whether certain members of the lung microbiota lead to the progress of pulmonary diseases or protect the organism [4,21]. 

Lactic acid bacteria (LAB) are well known for their immunomodulatory effects, and particular strains belonging to the genus Lactobacillus may have health-promoting effects and are denoted as probiotics [22]. A vast number of studies showed that LAB can have a wide range of immunomodulatory activities from immunostimulatory to anti-inflammatory activity [23]. Western Balkan countries (WBCs) have a long and rich experience in the production of artisanal dairy products, known for their diversity of LAB species and strains, with highly diverse health-promoting properties related to certain pathological conditions, e.g., diabetes, inflammatory pain, gut–brain axis-related diseases, aging, etc. [24,25,26,27,28]. However, up to now, the molecular mechanisms implicated in the effects of probiotics in respiratory epithelial cells have not been investigated though these species represent common members of healthy human lungs.

For this study, we selected 21 LAB strains from WBC artisanal dairy products together with human intestinal isolates from the collection at the Institute of Molecular Genetics and Genetic Engineering (IMGGE). In particular, the strains selected for this study were chosen based on preliminary results pointing to their antioxidant and anti-inflammatory activity, with the aim to select the best probiotic candidates that could be used in the prevention or treatment of inflammatory lung diseases. To the best of our knowledge, the present study, for the first time, investigates the immunomodulatory abilities of 21 natural LAB strains in LPS-stimulated human bronchial epithelial cells. We showed that LAB strains have an immunostimulatory effect, as well as an anti-inflammatory effect reducing the expression of pro-inflammatory mediators such as *IL-1β*, *IL-6*, *IL-8*, *TNF*, and *monocyte chemoattractant protein-1* (*MCP-1*). Additionally, we demonstrated that two LAB strains are able to attenuate the LPS-induced activity of NF-κB and MAPK signaling cascade resulting in the reduction of pro-inflammatory cytokine and chemokine expressions in bronchial epithelial cells.

## 2. Results

### 2.1. Safety Assessment of LAB Strains

In this study, 21 LAB strains were chosen from the IMGGE collection in order to evaluate their immunomodulatory properties in LPS-induced BEAS-2B cells. The strains belong to the: *Lactobacillus plantarum* (6), *Lb. paraplantarum* (1), *Lb. paracasei* subsp. *paracasei* (3), *Lb. helveticus* (1), *Lb. brevis* (2), *Lb. rhamnosus* (2), *Lactococcus lactis* subsp. *lactis* (4), *Lc. lactis* subsp. *cremoris* (1), and *Streptococcus thermophilus* (1) species. LAB strains and their sources are listed in Table 1. 

A safety evaluation of the LAB strains was performed by minimal inhibitory concentration (MIC) determination of bacterial antimicrobial susceptibility and an analysis of hemolytic and gelatinase activities. The results reveal that none of the strains exhibited hemolytic and/or gelatinase activity (data not shown). Moreover, in line with the European Food Safety Authority (EFSA) recommendations, the susceptibility of the strains to nine antibiotic groups was evaluated [40]. All of the LAB strains were susceptible to the tested antibiotics, and thus all of them have a qualified presumption of safety (QPS) status as shown in Table 2.

### 2.2. The Cytotoxicity

**Figure 1:**
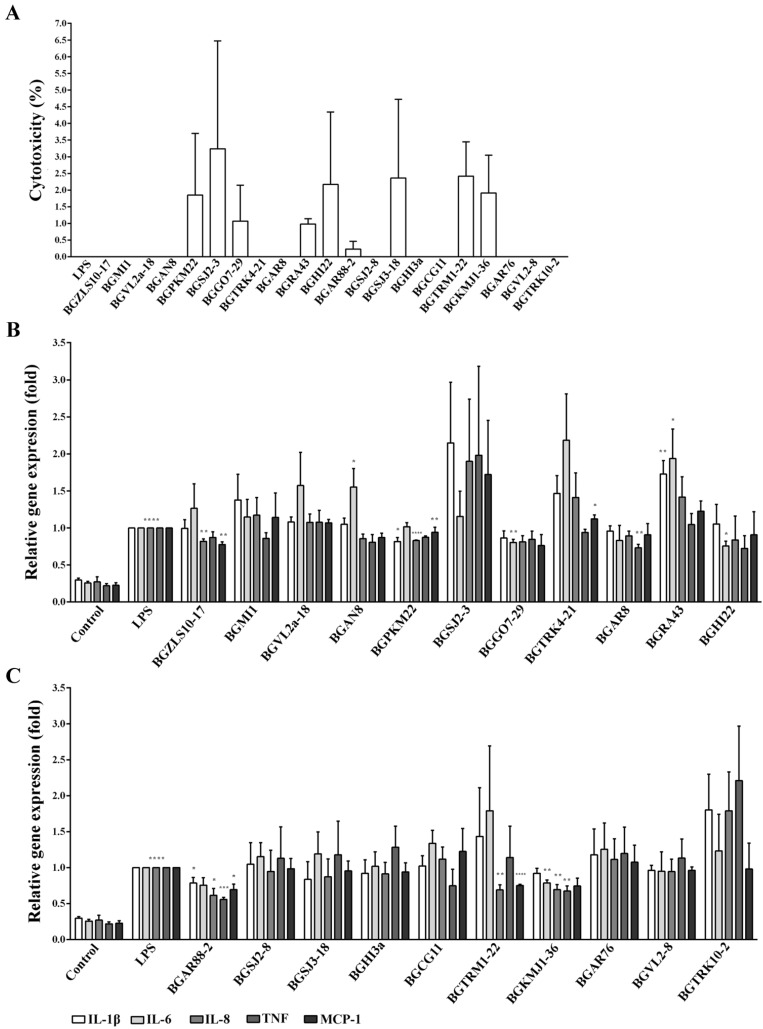
Cytotoxicity and the effect of 21 lactic acid bacteria (LAB) strains on the expression of pro-inflammatory genes in BEAS-2B cells. (**A**) Cells were treated with 21 LAB strains for two hours or lipopolysaccharide (LPS) (100 ng/mL) for 4 h, and the cytotoxicity was measured by lactate dehydrogenase (LDH) assay. (**B**) and (**C**) BEAS-2B cells without treatment (control) or treated with LPS served as experimental controls. Cells were pre-treated with 21 LAB strains for two hours and then treated with LPS for 4 h. The expression of *interleukin (IL)-1β, IL-6, IL-8, tumor necrosis factor (TNF),* and *monocyte chemoattractant protein 1 (MCP-1)* was determined by qRT-PCR. *Glyceraldehyde 3-phosphate dehydrogenase* (*GAPDH*) was used as endogenous control for normalization. The gene expression was calculated by ΔΔCT method relative to LPS. The results represent data from three independent experiments. Values are expressed as mean ± standard error of mean (SEM). Treatment with LPS is compared to control and treatment with LAB and LPS to LPS. Asterisks indicate significant differences when compared to LPS * *p* < 0.05, ** *p* < 0.01, *** *p* < 0.001, and **** *p* < 0.0001.

The cytotoxicity was tested by measuring the level of released lactate dehydrogenase (LDH) from the BEAS-2B cells exposed to the LAB strains at a 1:10 ratio or LPS (100 ng/mL). Our results show that there was no cytotoxicity (≤5%) of the chosen LAB strains or LPS used in the treatments of BEAS-2B cells, as shown in Figure 1A. 

### 2.3. Immunomodulatory Effects of 21 LAB Strains on the Expression of Pro-Inflammatory Genes

The ability of the chosen LAB strains to modulate immune responses was primarily analyzed in the BEAS-2B cells exposed to the LAB strains and stimulated with LPS using quantitative real-time polymerase chain reaction (qRT-PCR). Treatment with LPS significantly induced the expression of *IL-1β, IL-6, IL-8, MCP-1,* and *TNF* genes (Figure 1B,C). A total of 8 out of the chosen 21 LAB strains showed the ability to reduce the expression of at least one of the analyzed pro-inflammatory genes. Significant decrease in the LPS-induced inflammatory response was observed when BEAS-2B cells were treated with the following LAB strains: *Lb. brevis* BGZLS10-17 for *IL-8* (0.82 ± 0.03; *p* < 0.01) and *MCP-1* (0.78 ± 0.04; *p* < 0.001); *Lb. plantarum* BGPKM22 for *IL-1β* (0.82 ± 0.06; *p* < 0.05), *IL-8* (0.83 ± 0.01; *p* = 0.0001), and *TNF* (0.87 ± 0.02; *p* < 0.005); *Lb. plantarum* BGGO7-29 for *IL-6* (0.80 ± 0.04; *p* < 0.01); *Lc. lactis* BGAR8 for *TNF* (0.73 ± 0.05; *p* < 0.01); *Lb. rhamnosus* BGHI22 for *IL-6* (0.76 ± 0.07; *p* < 0.05) (Figure 1B); *Lb. paracasei* BGAR88-2 for *IL-1β* (0.79 ± 0.08; *p* < 0.05), *IL-8* (0.61 ± 0.09; *p* < 0.05), *MCP-1* (0.70 ± 0.08; *p* < 0.05), and *TNF* (0.56 ± 0.03; *p* = 0.0005); *Lc. cremoris* BGTRM1-22 for *IL-8* (0.69 ± 0.07; *p* < 0.01) and *MCP-1* (0.75 ± 0.01; *p* < 0.0001); and *St. thermophilus* BGKMJ1-36 for *IL-6* (0.79 ± 0.05; *p* < 0.01), *IL-8* (0.69 ± 0.08; *p* < 0.05), and *TNF* (0.65 ± 0.08; *p* < 0.01) gene expression in comparison to treatment with LPS (Figure 1B,C). On the contrary, a significant increase in the LPS-induced inflammatory response was detected when the cells were treated with the strains: *Lb. plantarum* BGAN8 for *IL-6* (1.55 ± 0.25; *p* < 0.05); *Lc. lactis* subsp. *lactis* BGTRK4-21 for *MCP-1* (1.12 ± 0.05; *p* < 0.05); and *Lb. helveticus* BGRA43 for *IL-1β* (1.72 ± 0.18; *p* < 0.001) and *IL-6* (1.93 ± 0.40; *p* < 0.05) gene expression compared to the LPS treatment (Figure 1B). Accordingly, the LAB strains BGZLS10-17, BGPKM22, BGGO7-29, BGAR88-2, and BGKMJ1-36 that exhibited the highest anti-inflammatory effects were selected for further characterization and analysis of molecular mechanisms. 

### 2.4. Adhesion of Selected LAB Strains to BEAS-2B Cells

**Figure 2:**
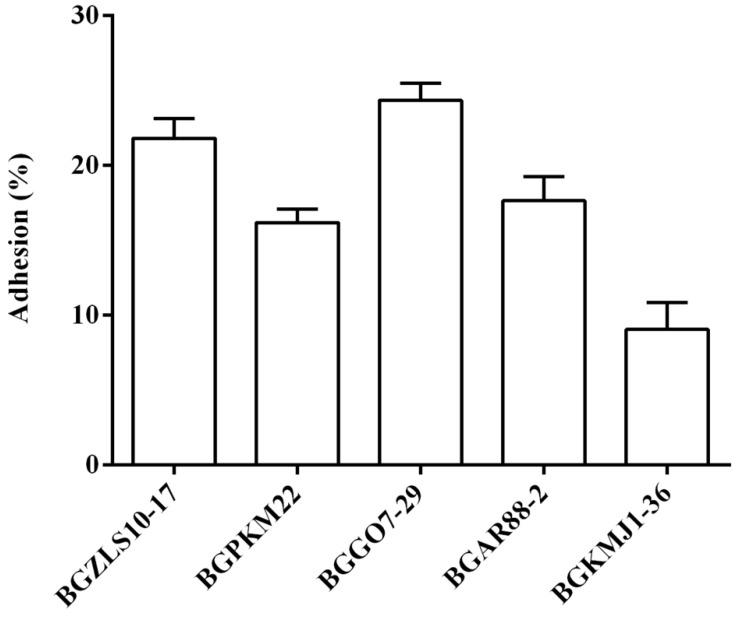
Adhesion of selected LAB strains on BEAS-2B cells. Cells were treated with LAB strains (1:10) for two hours. The levels of adhesion are expressed as % of LAB used for treatment. The results represent data from three independent experiments. Values are expressed as mean ± SEM.

The ability of the five selected LAB strains to adhere to the BEAS-2B cells was tested by the adhesion assay. According to our results, all selected LAB strains were able to adhere to the BEAS-2B cells though the levels of adhesion varied among the strains, as shown in Figure 2. The strain BGGO7-29 showed the highest adhesion (24.3 ± 1.1%), while BGKMJ1-36 showed the lowest adhesion (9.04 ± 1.8%) to BEAS-2B cells.

### 2.5. LAB Strains BGZLS10-17 and BGPKM22 Attenuated LPS-Induced Nuclear Translocation of NF-κB

**Figure 3:**
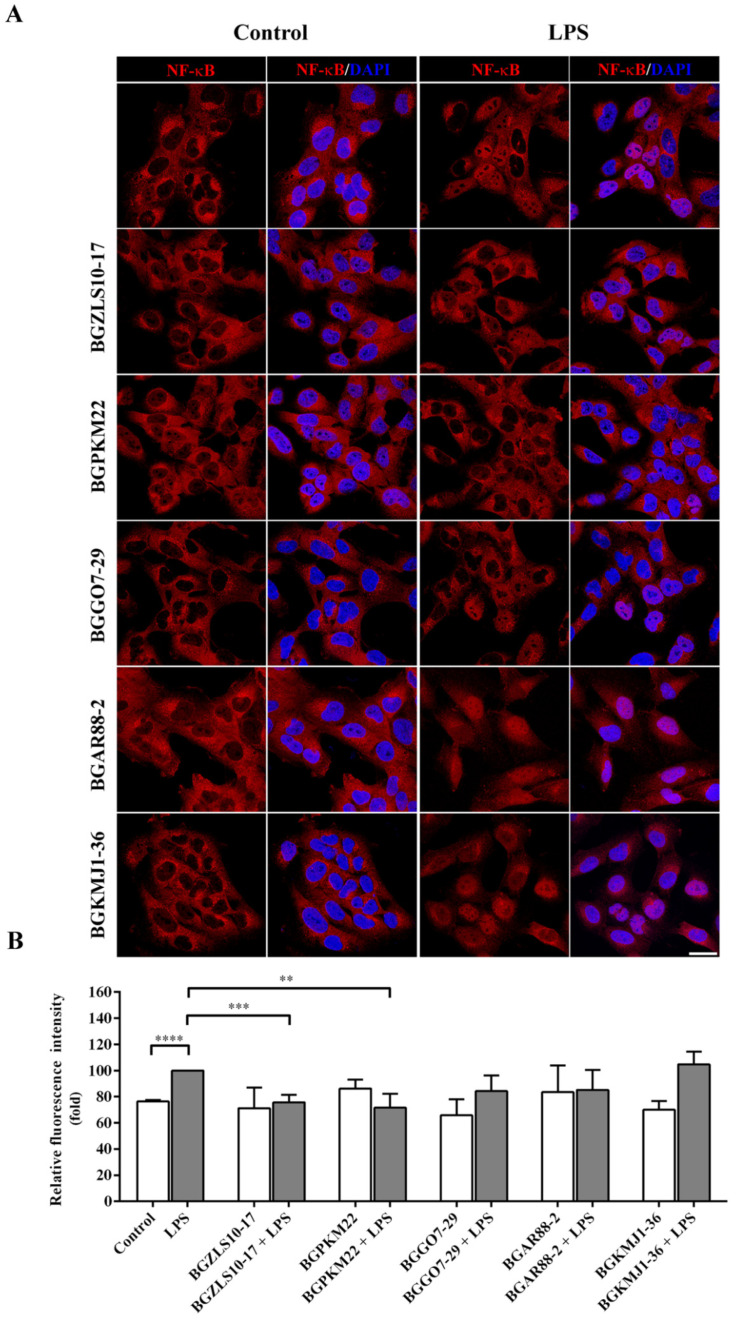
Nuclear translocation of nuclear factor (NF)-κB in BEAS-2B cells treated with selected LAB and LPS. BEAS-2B cells without treatment (control) or treated with LPS served as experimental controls. Cells were pre-treated with LAB strains for one hour and then treated with LPS for 30 min. (**A**) Immunostaining of NF-kB p65 nuclear translocation (red), nuclei were stained with diamino phenylindole (DAPI) (blue). Scale bar is 25 μm. (**B**) Mean fluorescence intensity of nuclear NF-kB is normalized to LPS. The results represent data from three independent experiments. Values are expressed as mean ± SEM. Treatment with LPS is compared to control, LAB to control, and LAB and LPS to LPS. Asterisks indicate significant differences ** *p* < 0.01, *** *p* < 0.001, and **** *p* < 0.0001.

In order to scrutinize the molecular mechanisms implicated in the anti-inflammatory effects of selected LAB, the ability of selected strains to affect the NF-κB nuclear translocation was tested. The activation of TLR4 by LPS leads to the translocation of the phosphorylated NF-κB transcription factor from the cytosol to the nucleus, consequently inducing the expression of pro-inflammatory genes. The BEAS-2B cells treated with LPS showed a significant increase in nuclear NF-κB compared to the control cells. The effects of the LAB strains on the LPS-induced nuclear translocation of NF-κB are shown in Figure 3A,B. 

The results of immunofluorescent staining and subsequent confocal microscopy show that the exposure of the BEAS-2B cells to selected LAB strains did not have a significant effect on the level of nuclear NF-κB (Figure 3A,B). However, *Lb. brevis* BGZLS10-17 and *Lb. plantarum* BGPKM22 showed the ability to attenuate the LPS-induced nuclear translocation of NF-κB. The level of nuclear NF-κB was significantly lower in cells treated with BGZLS10-17 (75.7 ± 5.8%, *p* = 0.0001) or BGPKM22 (71.6 ± 10.7%, *p* < 0.005) and LPS compared to cells treated with LPS only (Figure 3B). However, exposure of the BEAS-2B cells to the LAB strains BGGO7-29, BGAR88-2, and BGKMJ1-36 after LPS induction did not have a significant effect on the levels of nuclear NF-κB (Figure 3A,B).

Our results show that the LAB strains BGZLS10-17 and BGPKM22 block LPS-induced expression of pro-inflammatory genes by preventing NF-κB nuclear translocation in human bronchial epithelial cells. These two LAB strains able to block NF-κB signaling were further tested for the ability to interfere with the MAPK signaling cascade.

### 2.6. LAB Strains BGZLS10-17 and BGPKM22 Attenuate LPS-Induced Activation of p38, ERK1/2, and JNK MAPK Signaling 

Apart from activating the NF-κB transcription factor, TLR4 signaling also leads to the phosphorylation and activation of p38, ERK1/2, and JNK kinases [8]. The ability of the LAB strains BGZLS10-17 and BGPKM22, which showed a significant decrease in the nuclear translocation of NF-κB, to influence the activation of p38, ERK1/2, and JNK MAPK was tested in the BEAS-2B cells stimulated with LPS. 

**Figure 4:**
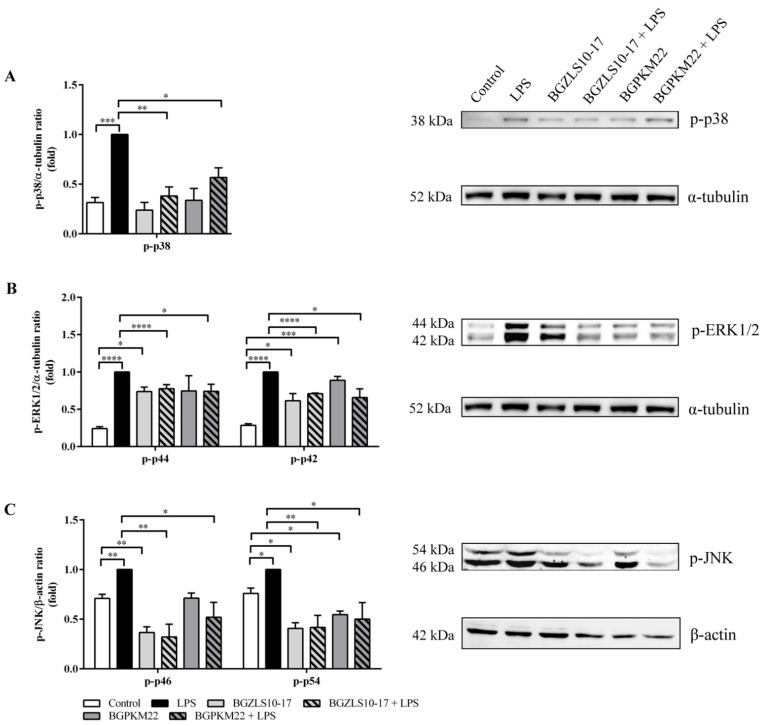
The activity of p38, extracellular signal-related kinases (ERK) 1/2, and c-Jun amino-terminal kinases (JNK) in BEAS-2B cells. BEAS-2B cells without treatment (control) or treated with LPS served as experimental controls. Cells were pre-treated with selected LAB for one hour and then treated with LPS for 20 min. Detection of (**A**) phospho-p38 (p-p38), (**B**) phospho-ERK1/2 (p-p44/p-p42), and (**C**) phospho-JNK (p-p46 and p-p54) by Western blot. Anti-α-tubulin and anti-β-actin were used as loading controls. Representative Western blots are shown on the right. The results represent data from three independent experiments. Values are expressed as mean ± SEM. Treatment with LPS is compared to control, LAB to control, and LAB and LPS to LPS. Asterisks indicate significant differences * *p* < 0.05, ** *p* < 0.01 *** *p* < 0.001, and **** *p* < 0.0001.

After exposure to LPS, the levels of phosphorylated p38, ERK1/2, and JNK noticeably increased in BEAS-2B cells compared with non-treated cells, as shown in Figure 4A–C. The levels of phosphorylated p38 were decreased in cells treated with the BGZLS10-17 (0.38 ± 0.09, *p* = 0.001) or BGPKM22 (0.57 ± 0.10, *p* < 0.05) and LPS in comparison to LPS (Figure 4A).

When the cells were exposed to the BGZLS10-17 or BGPKM22 strain, there was an increase in activated ERK1/2 (phospho-p44 and phospho-p42), suggesting immunostimulatory effects of both strains. However, the levels of phosphorylated ERK1/2 were decreased if the cells were treated with BGZLS10-17 (phospho-p44/p42 0.78 ± 0.05/0.71 ± 0.01, *p* < 0.0001) or BGPKM22 (phospho-p44/p42 0.74 ± 0.09/0.66 ± 0.12, *p* < 0.05) and LPS (Figure 4B) indicating the anti-inflammatory effects of the selected LAB strains. 

Our results show a decrease in activated JNK when the cells were treated with BGZLS10-17 or BGPKM22 strains for one or both isoforms of JNK, p46, and p54 (Figure 4C). The levels of phosphorylated JNK were also decreased in cells treated with the strain BGZLS10-17 (phospho-p46/p54 0.32 ± 0.13/0.42 ± 0.12, *p* < 0.01) or BGPKM22 (phospho-p46/p54 0.52 ± 0.15/0.50 ± 0.17, *p* < 0.05) and LPS, as shown in Figure 4C.

The obtained results show that both LAB strains, BGZLS10-17 and BGPKM22, are able to attenuate LPS-induced MAPK signaling that is responsible for pro-inflammatory gene expression via suppressing the activation of p38, ERK1/2, and JNK kinases.

## 3. Discussion

Chronic inflammatory lung diseases, such as COPD and asthma, are among the leading causes of severe illness and death, affecting more than 540 million people worldwide [41]. Smoking and respiratory infections are a major cause of the burden in chronic lung diseases, which are potentially preventable. Currently available anti-inflammatory pharmacological treatments have been ineffective in the suppression of lung inflammation, particularly in severe cases of lung diseases, which largely affect society and healthcare systems [41,42,43,44,45,46]. Thus, there is an urgent need for the application of novel approaches with alleviating effects on inflammation in chronic lung diseases. As the recruitment of inflammatory cells in the lungs is orchestrated via bronchial epithelial cells, modifying their inflammatory response could be an effective approach in the therapy of lung diseases [1]. As a target of environmental influences, microbiota, and bacterial pathogens, bronchial epithelial cells are also potent effectors that initiate and propagate inflammatory processes via the release of pro-inflammatory mediators, which further recruit and activate immune cells [1,2]. 

Although long considered as a sterile organ, lungs are characterized by a relatively low density of a complex microbial community of commensal bacteria. In contrast to the gastrointestinal tract, as an organ with the most studied bacterial community until now, the role of lung microbiota is relatively poorly investigated, and a comprehensive meta-analysis is still missing [47]. Recent studies demonstrated that lung microbiota change with the occurrence of pulmonary pathologies, such as inflammatory lung diseases, and also correlate with the severity and exacerbation of diseases implicating a direct contribution of the microbiota composition in the modulation of lung immunity [3,17,20]. The lung microbiota are characterized by a low density and a small number of bacteria, 2.2 × 10^3^ per cm^2^, due to the generally inhospitable lung environment for bacterial growth, and are constantly renewed and replaced by immigration, microaspiration, and elimination [4,18]. Gut microbiota are mainly involved in digestion, energy provision, synthesis of vitamins and other essential bioactive molecules, and maturation of the immune system. On the other hand, much less is known about the role of the lung microbiota. The growing body of evidence points to the gut–lung axis, suggesting the communication and interaction between gut and lung mucosal sites through the mucosal immune system as a wide organ [18,48,49]. It has been reported that oral probiotics reduce the risk of exacerbations and infections in chronic lung diseases [42,50]. For example, an intestinal commensal *Parabacteroides goldsteinii* has the ability to block NF-κB activation and inhibit lung inflammation by acting through the gut–lung axis [51]. Moreover, the lungs are exposed to multiple microbial challenges through different mechanisms, e.g., aerodigestive reflux and microaspiration, which may play an important role in the host immune response and immunological priming or contribute to a pathogenic immunological process relevant for disease development [52]. It has been demonstrated that the lung microbiome has a functional role in lung immunity and inflammation, but the components of this effects are still not clearly elucidated [16,53]. Moreover, there is a lack of data about the mechanisms of activity of potential probiotics that could be directly applied for the prevention or treatment of many lung disorders.

As natural inhabitants of healthy human lungs, LAB are also well-known for a variety of health-promoting effects including anti-inflammatory abilities, which are of particular interest in this study as they may express alleviating effects related to lung inflammation. Moreover, it has been reported that decreased bacterial diversity related to Firmicutes phyla is associated with the pathogenesis of emphysema and airway remodeling [21,50]. Due to the complexity of the lung microbiome, it is likely that various microbial products play an important role in lung immunity [52]. Moreover, it has been suggested that particular *Lactobacillus* spp. strains with probiotic properties could maintain physiological homeostasis of the lung and may provide a novel perspective for treating pulmonary diseases [16]. Probiotics can accomplish immunomodulatory effects by the inhibition of LPS binding to the CD14 receptor, but they also produce bioactive molecules or activate the expression of endogenous inhibitors of TLR4 signaling resulting in a reduction of pro-inflammatory cytokine production [8,51,54,55]. Thus, LAB strains capable of reducing the inflammatory response may be employed in the prevention or treatment of lung diseases characterized by diminished lung microbiota.

However, so far, immunomodulatory effects of LAB (Firmicutes) have not been investigated in the respiratory epithelial cells. Hence, the main objective of this study was to investigate the ability of natural LAB strains to modulate the inflammatory response in human bronchial epithelial cells used as an in vitro model system. Here, we showed that two LAB strains, *Lb. brevis* BGZLS10-17 and *Lb. plantarum* BGPKM22, possess anti-inflammatory properties by attenuating the activity of the NF-κB transcription factor and MAPK signaling cascade, consequently leading to lower pro-inflammatory gene expression. 

In this study, the effect of 21 natural LAB strains on the expression of LPS-induced pro-inflammatory genes in normal human bronchial epithelial cells was tested. The obtained results reveal that the strains exhibited a strain-specific effect, which is in line with previously published data [56].

In particular, the strains *Lb. plantarum* BGAN8, *Lc. lactis* subsp. *lactis* BGTRK4-21, and *Lb. helveticus* BGRA43 showed an immunostimulatory response increasing the expression of the *IL-1β, IL-6,* and *MCP-1* genes. On the contrary, several LAB strains exhibited an anti-inflammatory effect by reducing the expression of pro-inflammatory genes. In particular, the strains *Lb. brevis* BGZLS10-17, *Lb. plantarum* BGPKM22, BGGO7-29, *Lb. paracasei* BGAR88-2, and *St. thermophilus* BGKMJ1-36 showed significant anti-inflammatory abilities in LPS-stimulated BEAS-2B cells. Since LAB are lung commensals, the immune response of the lung may vary depending on the strains that come into the composition of lung microbiota. This opens up the possibility for the manipulation of lung microbiota, and LAB strains capable of reducing the inflammatory response, may be employed in the prevention or treatment of lung diseases characterized by diminished lung microbiota.

It is well-known that probiotics, such as *Lactobacillus* spp. and *Bifidobacterium* spp., can suppress inflammation by interfering with NF-κB and MAPK signaling checkpoints, which was mostly studied in intestinal epithelial or immune cells [8,51,54,55]. For the first time here, we show that LAB strains are capable to suppress inflammation by interfering with NF-κB and MAPK signaling in respiratory epithelial cells. We showed that two out of five LAB strains with anti-inflammatory abilities were capable to affect the activity of the main components of the TLR4 signaling cascade. We identified that two LAB strains, *Lb. brevis* BGZLS10-17 and *Lb. plantarum* BGPKM22, possess the capability to attenuate the LPS-induced nuclear translocation of NF-κB in bronchial epithelial cells resulting in a decrease in inflammatory gene expression. Additionally, we showed that these strains, BGZLS10-17 and BGPKM22, inhibit the activity of MAPK signaling components including p38, ERK1/2, and JNK, contributing to anti-inflammatory effects, too. The pro-inflammatory transcription factor NF-κB and MAPK kinases are activated in chronic lung diseases and implicated in the amplification of the inflammatory response in lungs [7,14,15]. They also have a prominent role in corticosteroid insensitivity and acute exacerbations of chronic lung diseases associated with significant morbidity and mortality [7]. Huge attempts have been made to develop drugs that can control the activity of NF-κB in chronic inflammatory lung diseases, such as the inhibitor of κB kinase (IKK) β inhibitors, but due to the critical role of NF-κB in the host’s defense against pathogens, there are concerns that inhibitors may increase the risk of infection [7]. Moreover, the application of inhibitors is usually followed by adverse side effects. In addition, there is a strong need for the development of MAPK inhibitors in order to reduce chronic lung inflammation and restore corticosteroid sensitivity. However, clinical trials have been largely disappointing [7]. Most anti-inflammatory drugs target turning off the pro-inflammatory arm of the inflammatory response, while approaches that turn on the body’s own anti-inflammatory mechanisms may be more precious. From that view, LAB would be ideal candidates. Probiotics can suppress inflammation by inhibiting various signaling pathways, and the use of probiotics targeting the NF-κB and MAPK pathways may have a profound anti-inflammatory effect [8]. Hence, the autochthonous strains, *Lb. brevis* BGZLS10-17 and *Lb. plantarum* BGPKM22, isolated from semi-hard cheese and artisanal sour milk that attenuate NF-κB and MAPK signaling may be employed for the alleviation or prevention of signs of chronic lung diseases.

The ability of the strains BGZLS10-17 and BGPKM22 to colonize human bronchial epithelial cells and to persist in the lungs, which is the prerequisite for their potential application as a probiotic for lungs, was tested in BEAS-2B cells [57]. The obtained results reveal that both strains are able to adhere to bronchial epithelial cells. In addition, antibiotic sensitivity and the absence of hemolytic and gelatinase activity were confirmed for these strains, indicating their QPS status and enabling their safety for application in humans as potential probiotics. 

In conclusion, this is the first study providing insight into the immunomodulatory effects of autochthonous natural LAB strains from Western Balkan countries in LPS-stimulated human bronchial epithelial cells. *Lb. brevis* BGZLS10-17 and *Lb. plantarum* BGPKM22, capable to inhibit prominent mediators of lung inflammation NF-κB and MAPK, could balance immune response and replenish diminished lung microbiota. These strains fulfil the demands for preclinical testing in an animal model in order to advance their application for the treatment of chronic lung diseases. Furthermore, a precise characterization of the LAB components responsible for anti-inflammatory effects will enable us to gain deeper insight into potential health benefit effects of *Lactobacillus* spp. strains.

## 4. Materials and Methods

### 4.1. Bacterial Strains, Media, and Growth Conditions

For the purpose of this study, 21 natural LAB strains were chosen from the IMGGE collection of microorganisms. The *Lactobacillus* strains were grown in De Man-Rogosa-Sharpe (MRS) medium (Merck GmbH, Darmstadt, Germany), while *Lactococcus* and *Streptococcus* strains were grown in M17 medium (Merck GmbH, Darmstadt, Germany) supplemented with 0.5% (*w*/*v*) glucose (GM17). Bacteria were grown at 30 or 37 °C under aerobic or anaerobic conditions depending on the strain. Solid medium was prepared by adding agar (1.7%, *w*/*v*) (Torlak, Belgrade, Serbia) to the broth medium. The strains were stored at −80 °C in an appropriate medium (GM17 or MRS) supplemented with 15% (*v*/*v*) glycerol. For the in vitro immunomodulatory assays, overnight cultures were harvested by centrifugation, washed with sterile phosphate-buffered saline (PBS), counted in a Petroff–Hausser counting chamber, and resuspended in Roswell Park Memorial Institute (RMPI) medium until use.

### 4.2. Antibiotic Susceptibility

Minimal inhibitory concentrations (MICs) were determined by microdilution testing according to the European Food Safety Authority (EFSA, Panel, 2018) guidance for bacterial antimicrobial susceptibility. Microbiological cut-off values (mg/L) of 9 antibiotics (ampicillin, vancomycin, gentamicin, kanamycin, streptomycin, erythromycin, clindamycin, tetracycline, and chloramphenicol) for each bacterial species were determined in accordance to the EFSA [40]. Determination of the MICs was performed by microdilution tests in Iso-Sensitest Broth (Oxoid, Hampshire, United Kingdom). The final coloni formin unit (CFU) per well was 5 × 10^6^. Cell density was monitored by OD_600_ measurements after 24 h of incubation at appropriate temperature in a microtiter plate reader (Tecan Austria GmbH, Grödig, Austria). The lowest concentration of antibiotic at which no growth of bacteria was detected was taken as MIC. Experiments were performed in triplicate.

### 4.3. Hemolytic and Gelatinase Activity Assays 

Hemolytic activity was determined on Columbia Blood Agar (Oxoid Limited, Hampshire, United Kingdom) containing 5% (*v*/*v*) defibrinated horse blood after 48 hours of incubation at 37 °C [58]. The phenotypic assay of gelatinase activity was determined on agar plates containing 3% (*w*/*v*) gelatin (Difco, Becton Dickinson, NJ, USA) [59].

### 4.4. Cell Culture

The normal human bronchial epithelial cell line BEAS-2B (ATCC CRL-9609) was cultured in RPMI medium (Gibco, ThermoFisher Scientific, Waltham, MA, USA) supplemented with 10% fetal bovine serum (FBS) (*Gibco*), 100 µg/mL streptomycin, and 100 U/mL penicillin (Sigma-Aldrich, St. Louis, MO, USA) at 37 °C and 5% CO_2_. For immunomodulatory assay, BEAS-2B cells with 80% confluence were pre-treated with natural LAB strains (Table 1) using 1:10 ratio in RPMI medium supplemented with 10% FBS without antibiotics for up to two hours. Subsequently, medium with LAB was removed, and cells were treated with 100 ng/mL of LPS (Sigma-Aldrich, St. Louis, MO, USA) for up to 4 h. All experiments were performed at least three times, independently.

### 4.5. Lactate Dehydrogenase (LDH) Release Assay

The cellular cytotoxicity was determined by measuring the activity of LDH from the culture medium. The activity of LDH was measured spectrophotometrically by CyQUANT LDH Cytotoxicity Assay Kit (ThermoFisher Scientific, Waltham, MA, USA), as per the manufacturer’s instructions. Briefly, 1.7 × 10^4^ cells per well in 96-well plate were seeded and treated with LAB, using a 1:10 ratio for two hours and LPS (Sigma-Aldrich, St. Louis, MO, USA), 100 ng/mL for 4 h. The activity of LDH was determined by measuring the absorbance at 490 nm and 680 nm, using a microtiter plate reader (Tecan Austria GmbH, Grödig, Austria). The cytotoxicity was calculated as the percentage relative to unstimulated cells.

### 4.6. Quantitative Real Time-PCR (qRT-PCR) Assay

A total of 2.5 × 10^5^ BEAS-2B cells per well were seeded, and the next day the cells were pre-treated with LAB in a 1:10 ratio for two hours. Afterwards, the medium was removed and replaced with fresh medium with LPS 100 ng/mL for 4 h. Total RNA was isolated from BEAS-2B cells using the RNeasy Plus Mini Kit (Qiagen, Hilden, Germany) according to the manufacturer’s instructions. RNA purity and concentration were determined by BioSpec-nano (Shimadzu Corporation, Kyoto, Japan).

cDNA was synthesized from 1 μg of total RNA using the High Capacity cDNA Reverse Transcription Kit (ThermoFisher Scientific, Waltham, MA, USA) as per the manufacturer’s instructions. TaqMan Gene Expression Assays (ThermoFisher Scientific, Waltham, MA, USA) for the *IL-1β, IL-6, IL-8, MCP-1, TNF,* and endogenous control, *glyceraldehyde 3-phosphate dehydrogenase* (*GAPDH*) gene, were used for RT-PCR on 7500 Real-Time PCR system (Applied Biosystems, Waltham, MA, USA) following manufacturer’s protocols. Relative quantification by ΔΔCT method was used to calculate gene expression relative to positive control (LPS), which was used as a calibrator. Results were analyzed using Applied Biosystems 7500 System Software.

### 4.7. Adhesion Assay

The ability of selected LAB to adhere to BEAS-2B cells was analyzed by adhesion assay according to Mushtaq et al. with minor modifications [60]. Overnight bacterial culture was washed twice with PBS solution, and the pellets were resuspended in RPMI media without antibiotics. A total of 2.5 × 10^5^ BEAS-2B cells, seeded the day before, were washed twice with PBS and treated with bacterial suspensions using a ratio of 1:10 (BEAS-2B cells: LAB). Following co-incubation for two hours at 37 °C and 5% CO_2,_ the cells were gently washed with PBS and lysed with 0.25% Trypsin–EDTA solution (Sigma-Aldrich, St. Louis, MO, USA). Serial dilutions of samples, before and after adhesion, were plated on appropriate agar plates. The adhesion was calculated as (%) = (CFU/mL adhered bacteria/CFU/mL added bacteria) × 100.

### 4.8. Immunocytochemistry

BEAS-2B cells were seeded on coverslips and pre-treated the next day with LAB in 1:10 ratio for one hour and treated with LPS 100 ng/mL for 30 min. The cells were fixed using 3% paraformaldehyde and 2% sucrose for 20 min at room temperature (RT) and permeabilized in 0.2% Triton X-100 for 10 min. Non-specific binding was blocked in 10% normal goat serum (Sigma-Aldrich, St. Louis, MO, USA) and 1% bovine serum albumin (BSA) (Serva, Heidelberg, Germany) in PBS for one hour at RT. An anti-NF-kB p65 antibody (Alexa Fluor^®^ 594, Abcam) was diluted 1:400 in 1% BSA in PBS and applied for one hour at RT. Coverslips were washed 3 times for 10 min in 0.2% Tween 20 in PBS. Nuclei were stained with 0.1 mg/mL diamino phenylindole (DAPI, Sigma-Aldrich, St. Louis, MO, USA). Coverslips were mounted on to the glass slides using Fluorescent Mounting Medium (Dako, Via Real Carpinteria, CA, USA). 

### 4.9. Image Acquisition and Analysis

Fluorescence images were captured on a Leica SP8 confocal laser scanning system (Leica Microsystems, Wetzlar, Germany). The AF594 labelled antibody was excited with an OPSL 552 laser, while DAPI was excited with 405 diode laser. DAPI (excitation 405 nm, emission 400–450 nm) and AF594 (excitation 590 nm, emission 618 nm) fluorescence were captured using sequential acquisition to give separate image files for each. A pin hole of 1 Airy (95.5 μm), scan speed of 200 Hz and 4 frame averaging, was used. Photomultiplier tube gain and offset were adjusted to give sub-saturating fluorescence intensity with optimal signal to noise ratio. Image acquisition settings were the same for all examined samples in order to compare fluorescence intensities. Six Z stacks in the range of 1.47 µm were acquired. 

Quantification of NF-κB nuclear translocation was performed by image analysis using LAS X software [61]. Mean fluorescence intensity was measured on the maximal projections. Fluorescence was quantified in a minimum of 100 nuclei per sample, in 3–5 randomly chosen fields. Quantification was performed with the histogram tool and polygonal region of interest (ROI), by two independent measurements. The nuclear ROIs were defined on DAPI channel. Quantitative fluorescence data were exported from LAS X-generated data into Microsoft Excel software for further analysis and presentation. Nuclear NF-κB was expressed as % of increase in nuclear fluorescent signal compared to 100% set for the cells treated with LPS.

### 4.10. Western Blotting

A total of 10^5^ BEAS-2B cells were seeded, and the next day the medium was changed to serum-free RPMI for 24 hours. The cells were pre-treated with LAB in a 1:10 ratio for one hour and treated with LPS 100 ng/mL for 20 min. The cells were washed with ice-cold PBS and lysed with Radio Immuno Precipitation Assay buffer (50 mM Tris-HCl pH 7.4; 150 mM NaCl; 1% NP-40; 0.25% sodium deoxycholate) containing protease inhibitor cocktail tablets (Roche, Basel, Switzerland) and 1 mM phenylmethylsulphonyl fluoride (Sigma-Aldrich, St. Louis, MO, USA), for 30 min on ice. The total protein concentrations were determined using the BCA protein assay kit (ThermoFisher Scientific, Waltham, MA, USA). Equal amounts of proteins (15 μg) were separated by 12.5% sodium dodecyl-sulfate polyacrylamide gel electrophoresis (SDS-PAGE). Electrophoresed proteins were transferred from the gel to a 0.2 μm nitrocellulose membrane (GE Healthcare) using a Bio-Rad Mini *trans*-blot system (Bio-Rad, Hercules, CA, USA). Immunoblots were blocked in a 5% non-fat dry milk in PBS-Tween (50 mM Tris-HCl, pH 7.4; 150 mM NaCl, and 0.05% Tween-20) overnight at 4 °C. After that, the immunoblots were incubated with primary antibodies anti-phospho-p38 MAPK (Thr180/Tyr182), anti-phospho-p44/42 MAPK (Erk1/2) (Thr202/Tyr204), and anti-phospho-JNK (Thr183/Tyr185) (Cell Signaling Technology, Danvers, MA, USA) and anti-α-tubulin and anti-β-actin as a loading controls (Cell Signaling Technology, Danvers, MA, USA) for two hours at RT. The membranes were subsequently washed and incubated with appropriate horseradish peroxidase (HRP) conjugated secondary antibody (ThermoFisher Scientific, Waltham, MA, USA) for one hour at RT. Proteins were detected by enhanced chemiluminescence Immobilon Western (Merck Millipore, Burlington, MA, USA).

### 4.11. Statistical Analysis 

The data are expressed as mean ± standard effort of mean (SEM). The statistical significance of the differences between treatment and control was tested by Student’s t-test. A *p*-value of less than 0.05 was considered significant. Statistical analysis was carried out, and graphs were prepared by using the GraphPad Prism 6 software.

## Figures and Tables

**Table 1:** The list of bacterial strains and their sources used in this study.

Bacterial Strain	Source	References
*Lactobacillus brevis* BGZLS10-17	10-day-old semi-hard cheese	[29,30]
*Lactobacillus plantarum* BGMI1	White cheese	IMGGE collection
*Lactobacillus plantarum* BGVL2a-18	15-day-old raw goat milk cheese	[31]
*Lactobacillus plantarum* BGAN8	Artisanal soft cheese	IMGGE collection
*Lactobacillus plantarum* BGPKM22	Artisanal sour milk	IMGGE collection
*Lactobacillus plantarum* BGSJ2-3	White cow cheese	IMGGE collection
*Lactobacillus plantarum* BGGO7-29	60-day-old white pickled cheese	[32]
*Lactococcus lactis* subsp. *lactis* BGTRK4-21	Sweet kajmak	[33]
*Lactococcus lactis* subsp. *lactis* BGAR8	5-day-old goat cheese	[34]
*Lactobacillus helveticus* BGRA43	Human intestinum	[35,36,37]
*Lactobacillus rhamnosus* BGHI22	Human intestinum, neonatus	IMGGE collection
*Lactobacillus paracasei* BGAR88-2	5-day-old goat cheese	[34]
*Lactobacillus paracasei* BGSJ2-8	White cow cheese	[38]
*Lactobacillus rhamnosus* BGSJ3-18	White cow cheese	[39]
*Lactobacillus brevis* BGHI3a	Human intestinum, neonatus	IMGGE collection
*Lactobacillus paraplantarum* BGCG11	Old full-fat cheese	IMGGE collection
*Lactococcus lactis* subsp. *cremoris* BGTRM1-22	Sweet cream	[33]
*Streptococcus thermophilus* BGKMJ1-36	Artisanal sour milk	[26]
*Lactobacillus paracasei* BGAR76	5-day-old goat cheese	[34]
*Lactococcus lactis* subsp. *lactis* BGVL2-8	5-day-old raw goat milk cheese	[31]
*Lactococcus lactis* subsp. *lactis* biovar. diacetylactis BGTRK10-2	Sweet kajmak	[33]

IMGGE—Institute of Molecular Genetics and Genetic Engineering.

**Table 2:** Minimal inhibitory concentrations (MICs) of nine antibiotics according to EFSA (2018).

	Antibiotic with the MIC (ug/mL)								
Strains	Amp	Van	Gen	Kan	Str	Ery	Cln	Tet	Chl
*Lb. plantarum*									
BGMI1	<1	n.r.	8	32	n.r.	<1	2	16	4
BGVL2a-18	1	n.r.	4	16	n.r.	<1	2	16	4
BGAN8	1	n.r.	8	16	n.r.	<1	2	16	4
BGPKM22	<1	n.r.	4	16	n.r.	<1	2	16	4
BGSJ2-3	1	n.r.	4	32	n.r.	<1	2	16	4
BGGO7-29	1	n.r.	8	16	n.r.	<1	2	16	4
BGCG11	<1	n.r.	4	8	n.r.	<1	1	8	1
*Lb. brevis*									
BGZLS10-17	1	n.r.	4	32	32	<1	2	4	2
BGHI3a	1	n.r.	4	32	16	<1	2	4	2
*Lb. helveticus*									
BGRA43	<1	1	4	4	4	<1	1	2	1
*Lb. rhamnosus*									
BGHI22	2	n.r.	8	32	16	<1	2	2	2
BGSJ3-18	2	n.r.	8	16	16	<1	2	2	2
*Lb. casei*									
BGSJ2-8	2	n.r.	16	16	32	<1	2	2	2
BGAR76	1	n.r.	8	32	32	<1	2	2	2
BG88-2	1	n.r.	8	32	32	<1	2	2	2
*St. thermophilus*									
BGKMJ1-36	<1	<0.5	16	n.r.	32	1	0.5	2	1
*Lc. lactis*									
BGTRK4-21	<1	2	16	32	16	<1	<1	2	4
BGAR8	<1	2	16	32	16	<1	<1	2	4
BGAR76	1	2	16	32	16	<1	<1	2	2
BGVL2-8	<1	2	8	32	8	<1	<1	2	2
BGTRK10-2	<1	2	16	32	16	<1	<1	2	2

n.r.—not required; Amp—ampicillin; Van—vancomycin; Gen—gentamicin; Kan—kanamycin; Str—streptomycin; Ery—erythromycin; Cln—clindamycin; Tet—tetracycline; Chl—chloramphenicol.

## Data Availability

The data presented in this study are available on request from the corresponding author.

## Abbreviations

BEAS-2B: Normal human bronchial epithelial cell line; BSA: Bovine serum albumin; CF: Cystic fibrosis; CFU: Colony forming units; COPD: Chronic obstructive pulmonary disease; DAPI: 4′,6-Diamidino-2-phenylindole dihydrochloride; EFSA: European Food Safety Authority; ERK: Extracellular signal-regulated kinase; FBS: Fetal bovine serum; HRP: Horseradish peroxidase; IKK: Inhibitor of κB kinase; IL: Interleukin; IMGGE: Institute of Molecular Genetics and Genetic Engineering; JNK: c-Jun NH 2-terminal kinase; LAB: Lactic acid bacteria; Lb. brevis: Lactobacillus brevis; Lb. helveticus: Lactobacillus helveticus; Lb. paracasei: Lactobacillus paracasei; Lb. paraplantarum: Lactobacillus paraplantarum; Lb. plantarum: Lactobacillus plantarum; Lb. rhamnosus: Lactobacillus rhamnosus; Lc. Lactis: Lactococcus lactis; LDH: Lactate dehydrogenase; LPS: Lipopolysaccharides; MAPK: Mitogen-activated protein kinase; MCP: Monocyte chemoattractant protein; MIC: Minimal inhibitory concentration; MRS: De Man Rogosa Sharpe; NF-κB: Nuclear factor-κB; PAMP: Pathogen-associated molecular pattern; PBS: Phosphate-buffered saline; PRR: Pattern recognition receptor; QPS: Qualified presumption of safety; qRT-PCR: quantitative Real time-polymerase chain reaction; ROI: Region of interest; RPMI: Roswell Park Memorial Institute; RT: Room temperature; SDS-PAGE: Sodium dodecyl-sulfate polyacrylamide gel electrophoresis; SEM: Standard effort of mean; St. thermophilus: Streptococcus thermophilus; TLR: Toll-like receptor; and TNF: Tumor necrosis factor; WBC: Western Balkan countries.
